# Serum Glutamate in Dry Eye Disease: Associations with Symptoms and Clinical Signs

**DOI:** 10.3390/diagnostics16091338

**Published:** 2026-04-29

**Authors:** Brinna Desai, Rohit Muralidhar, Loralei Parchejo, Jonghoon Chang, Luisa Saccaro, Kristina Aenlle, Raquel Goldhardt, Anat Galor

**Affiliations:** 1 Bascom Palmer Eye Institute, Department of Ophthalmology, University of Miami Miller School of Medicine, Miami, FL 33136, USA; 2 College of Osteopathic Medicine, Nova Southeastern University, Fort Lauderdale, FL 33328, USA; 3 South Florida VA Foundation for Research and Education, Inc., Miami, FL 33125, USA; 4 Surgical and Research Services, Miami Veterans Administration Medical Center, 1201 NW 16th St., Miami, FL 33125, USA; 5 Institute for Neuro-Immune Medicine, Nova Southeastern University, Fort Lauderdale, FL 33328, USA

**Keywords:** dry eye disease, glutamate, neurotoxicity, corneal nerves, ocular surface disease

## Abstract

**Background/Aims**: Glutamate is the primary excitatory neurotransmitter in the central nervous system, and elevated levels have been implicated in neurotoxicity. Corneal nerves are integral to ocular surface health and abnormalities can contribute to dry eye disease (DED). This study evaluates the relationship between serum glutamate levels, DED signs and symptoms, and corneal nerve parameters. **Methods**: A total of 124 veterans evaluated at the Miami Veterans Affairs Eye Clinic were included. Participants completed standardized questionnaires assessing ocular surface symptoms and pain, underwent comprehensive ocular surface examination and in vivo confocal microscopy, and provided blood samples for serum glutamate analysis. **Results**: Mean age was 55.67 ± 4.59 years; 90.3% were male, 55.6% White, and 35.5% Hispanic. Serum glutamate ranged from 0.26 to 3.16 µM/µL (mean 1.22 ± 0.57 µM/µL). Glutamate levels were not associated with DED symptoms as assessed by standardized questionnaires. Higher glutamate levels were linked with DED signs including reduced tear production (right eye: r = −0.05, *p* = 0.55; left eye: r = −0.25, *p* = 0.01) and increased corneal staining (right eye: r = 0.10, *p* = 0.29; left eye: r = 0.20, *p* = 0.03). Most notable were associations between elevated glutamate and corneal nerve health, including reduced corneal sensation (right eye: r = −0.20, *p* = 0.03; left eye: r = −0.18, *p* = 0.047) and decreased corneal nerve fiber width (r = −0.23, *p* = 0.01). **Conclusions**: Our findings support an association between systemic neurochemical status, specifically circulating glutamate, and ocular surface and corneal nerve health.

## 1. Introduction

Dry eye disease (DED) is a heterogeneous disorder of the ocular surface leading to disruption of tear film homeostasis in combination with ocular symptoms. Contributing mechanisms include tear film instability and hyperosmolarity, inflammation, and neurosensory abnormalities [1]. The latter is particularly relevant as the cornea possesses a dense sensory nerve network that detects thermal, mechanical, and chemical stimuli, initiating reflexes such as blinking and tearing, as well as sensations of dryness and discomfort [2]. This corneal sensory innervation arises from the ophthalmic branch of the trigeminal nerve and courses through the nasociliary nerve and long ciliary branches before entering the cornea as nerve fibers [2,3]. Corneal nerves may become dysfunctional through various mechanisms—including chronic inflammation [4,5], infection [6], or surgical injury [7], among others—resulting in variable degrees of nerve dysfunction that contribute differently to DED manifestations. The frequent discordance between DED symptoms and measurable tear film abnormalities [8] further supports a role for nerve dysfunction as a key contributing mechanism to disease.

Given the emerging role of nerve dysfunction in DED, molecular factors that regulate nerve function, including neurotransmitters, have gained increasing attention. Glutamate, one of the primary excitatory neurotransmitters in the central nervous system, is one such molecule as it is essential for neural transmission [9]. However, excessive extracellular levels can initiate excitotoxicity, a pathological process characterized by uncontrolled continuous depolarization of neurons [10]. Excitotoxicity has been implicated in structural neuronal injury and nerve cell death [10]. In addition to neuronal loss, glutamate excitotoxicity has been associated with alterations in nerve morphology, including changes in axonal structure and synaptic architecture, indicating that glutamate may affect neuronal processes independently of cell death [11]. Moreover, the presence of glutamate receptors in corneal sensory afferents supports the involvement of glutamatergic signaling in corneal nerve function [12]. Given the dense corneal sensory network and its reliance on intact neural signaling to maintain ocular surface homeostasis, dysregulation of glutamate may have impactful implications in DED and corneal nerve function. The potential relevance of glutamate to ocular surface disease has been explored in prior studies. In one study, glutamate concentration (μM) in tear fluid from patients with severe ocular surface disease (i.e., Stevens–Johnson syndrome, chemical injury, thermal burn, stem cell deficiency; *n* = 18) was significantly higher than those with ocular disorders not primarily affecting the ocular surface (e.g., granular dystrophy, band-shaped keratopathy, keratoconus; *n* = 15), ~75–90 versus ~10 μM, *p* < 0.01 [13]. On the other hand, in 55 individuals with clinically diagnosed DED, lower tear glutamate (measured by spectroscopy) levels were noted compared to 35 controls (0.027 ± 0.004 vs. 0.031 ± 0.01 absorbance units, *p* = 0.001) [14].

Beyond ocular surface dysfunction, glutamate has been implicated in neuronal injury across ocular and systemic neurodegenerative conditions, supporting its role as a potential mediator of disease pathology. In one study, a twofold increase in intravitreal glutamate was observed in glaucoma patients compared to controls sampled at the time of cataract surgery (23.1 ± 4.6 vs. 10.0 ± 5.1 µmol/L, *p* < 0.001; *n* = 26 vs. 21) [15], suggesting glutamate-mediated excitotoxicity may contribute to retinal ganglion cell injury and optic nerve damage in glaucoma. Systemic alterations in glutamate have also been reported in ocular disease. Serum glutamate levels were found to be 18% higher in patients with diabetic retinopathy compared to those with type 2 diabetes without retinopathy (fold change = 1.18, *p* = 0.007, *n* = 69 per group) [16], implicating glutamate in retinal neurovascular pathology. Elevated circulating glutamate has also been linked to neuronal injury in non-ocular neurodegenerative disease, with higher plasma levels correlating with larger ischemic lesion volumes on MRI at 72 h in acute stroke (r = 0.71, *p* < 0.001; *n* = 197) [17], and significantly increased serum concentrations reported in patients with multiple sclerosis compared to controls (1.32 ± 0.54 vs. 0.87 ± 0.34 nmol/mL, *p* < 0.001; *n* = 55 vs. 25) [18]. Together, these findings support a broader role for systemic glutamate in neural injury in and outside the eye, raising the possibility of its relevance in DED. However, gaps in the literature remain, including whether circulating glutamate levels are associated with ocular phenotypes in DED, including symptoms, signs of tear dysfunction, and/or corneal nerve abnormalities. The objective of this study was to evaluate whether serum glutamate levels are associated with (1) clinical signs and symptoms of dry eye disease and (2) corneal nerve structure and function in a cross-sectional cohort.

## 2. Materials and Methods

### 2.1. Study Population

A cross-sectional analysis was performed using 124 veterans who were seen at the Miami Veterans Affairs (VA) Eye Clinic between October 2020 and July 2022. Inclusion criteria required Gulf War–era veteran status (as part of a larger eye health study in this population) and normal external ocular anatomy, including eyelids, conjunctiva, and cornea. Exclusion criteria included the use of any ocular medications with the exception of artificial tears, presence of significant ocular diseases which could affect ocular surface health (such as glaucoma, prior retinal surgery, pterygium), systemic autoimmune conditions associated with DED (such as Sjögrens or graft-versus-host disease), and medical or psychiatric conditions that could interfere with completion of study procedures or questionnaires. The study was approved by the Miami Veterans (VA) Institutional Review Board and all participants provided informed consent. Study procedures were conducted in accordance with the ethical standards of the Declaration of Helsinki and the requirements of the U.S. Health Insurance Portability and Accountability Act (HIPAA). The study protocol was standardized across participants and conducted in the following order on the same day: (1) informed consent, (2) collection of demographic information and administration of standardized questionnaires, (3) blood collection for serum glutamate measurement, (4) comprehensive ocular examination, and (5) in vivo confocal microscopy (IVCM).

### 2.2. Data Collection

Participants self-reported medical and ocular history, demographic information, and use of medications. They also completed standardized questionnaires to evaluate ocular surface symptoms. Symptoms of dry eye were measured using the Ocular Surface Disease Index (OSDI, range 0–100) [19] and 5-Item Dry Eye Questionnaire (DEQ5, range 0–22) [20]. These forms allowed for the evaluation of various symptoms including ocular discomfort, visual function, and environmental triggers. Ocular pain was evaluated using a Numerical Rating Scale (NRS, range 0–10, intensity of eye pain “right now,” “average over the last week,” and “worst over the last week” [21,22] and with the Neuropathic Pain Symptom Inventory, modified for Eye pain (NPSI-Eye, total score: range 0–100; sub-score range 0–10) [23]. Other health indicators were measured using the Patient Health Questionnaire (PHQ-9) for depression (total score 0–27) [24] and the Modified Fatigue Impact Scale (MFIS) for fatigue (total score 0–84) [25].

### 2.3. Serum Glutamate Collection

Peripheral blood samples obtained by venipuncture were further analyzed for glutamate concentration using a fluorometric Glutamate Assay Kit (Abcam, ab138883, Cambridge, UK). Samples were run following the manufacturer’s protocol.

### 2.4. Ocular Examination

Patients underwent examination of ocular signs in the following order: (1) Ocular surface inflammation was evaluated using InflammaDry testing with qualitative measurement of matrix metalloproteinase 9 (MMP-9) graded as 0 = none, 1 = mild, 2 = moderate, and 3 = severe, based on the intensity of the pink stripe [26]. MMP-9 measurements were not obtained in a subset of subjects due to intermittent supply limitations during the study period. (2) Corneal sensation was assessed with a cotton-tipped applicator and graded as 0 = absent, 1 = reduced, 2 = normal, or 3 = increased [27]. (3) Tear stability was measured by tear break-up time (TBUT) following instillation of 5 μL of fluorescein dye. Three readings were obtained and averaged with values ≤ 5 s considered abnormal. (4) Corneal epithelial integrity was evaluated using fluorescein corneal staining graded according to the National Eye Institute (NEI) scale [28]. Five corneal regions were scored from 0 to 3 (total range: 0–15), where 0 indicates no staining and 3 represents severe, confluent staining. A total score ≥ 3 (out of 15) was considered abnormal. (5) Pain intensity was measured using a 0–10 NRS before and 30 s after instillation of 10 µL of 0.5% proparacaine hydrochloride with a score of 0 denoting no pain, and 10 representing the worst imaginable pain. (6) Tear production over 5 min was measured using the Schirmer test after application of topical anesthesia (range: 0–35 mm), with values ≤ 5 mm considered abnormal. (7) Intraocular pressure (IOP) was measured with a Tono-Pen XL^®^ applanation tonometer (Reichert, Depew, NY, USA). The sequence of ocular testing was chosen to minimize interference between measures, based on previously studied data [29,30,31].

### 2.5. Confocal Analysis

Assessment of corneal nerve features was performed using IVCM with the Rostock Cornea Module integrated into the Heidelberg Retina Tomograph III (Heidelberg Engineering, Heidelberg, Germany) [32]. Images were analyzed with a validated automated nerve analysis software (ACCMetrics Corneal Nerve Fiber Analyser, Version 2; University of Manchester, Manchester, UK) [33]. Qualitative review of included images was independently conducted by a masked grader to allow for unbiased assessment. Six confocal images were selected from each eye for analysis.

The automated analysis provided metrics for multiple parameters including: corneal nerve fiber density (CNFD; fibers/mm^2^), defined as the number of main nerve fibers per square millimeter; corneal nerve fiber length (CNFL; mm/mm^2^), defined as the total length of all main and branch fibers per square millimeter; corneal nerve branch density (CNBD; branches/mm^2^), defined as the number of branch points per square millimeter; corneal nerve fiber area (CNFA; mm^2^/mm^2^), define as the total area occupied by nerve fibers; corneal nerve fiber width (CNFW; mm/mm^2^), representing the mean fiber width; and corneal total branch density (CTBD; branches/mm^2^), representing the total number of branch points per square millimeter [34,35]. The corneal nerve fractal dimension was also evaluated as a quantitative index of corneal nerve architectural complexity. This metric has been applied to characterize structural alterations associated with neurodegenerative and ocular surface disorders [36]. For each parameter above, the minimum, maximum, and mean values were derived from the outputs of the six analyzed images from each eye.

### 2.6. Statistical Analysis

Statistical analyses were performed using SPSS, Version 31.0 (IBM Corp., Armonk, NY, USA). Demographic and clinical data were derived and compiled using descriptive statistics functions. Univariate correlations (*t* tests, Chi Square, Pearson and Spearman correlations) were performed to evaluate relationships between serum glutamate and clinical metrics, followed by multivariable modeling. A *p*-value < 0.05 was considered statistically significant. The sample size of 124 was determined to be adequate for detecting associations of moderate effect size [37].

## 3. Results

### 3.1. Study Population

Our population consisted of 124 individuals. The mean age of participants was 55.67 ± 4.59 years; 90.3% (*n* = 112) self-identified as male, 55.6% (*n* = 69) as White, and 35.5% (*n* = 44) as Hispanic (Table 1).

Most individuals had clinically significant ocular symptoms based on the OSDI (33.75 ± 23.43) and DEQ5 (8.93 ± 4.63), with 75.8% and 77.5% having moderate or greater symptoms on the two questionnaires (OSDI ≥ 13, DEQ5 ≥ 6). Beyond ocular symptoms, a majority of individuals displayed symptoms of additional health indicators including depression (74.3%) and fatigue (53.3%), as measured by the PHQ-9 (≥5) and MFIS (≥38), respectively.

On clinical examination, objective signs of ocular surface disease were generally mild. Most individuals demonstrated minimal ocular surface inflammation, with preserved tear production and tear stability, as reflected by Schirmer scores and TBUT values within normal ranges. Corneal sensation testing similarly showed predominantly normal sensation, with a minority of participants exhibiting either hypoesthesia (right eye: 11.3%, left eye: 14.5%) or hyperesthesia (right eye: 6.5%, left eye: 9.7%). Additionally, a minority of patients who reported pain before topical anesthesia continued to experience pain following anesthetic administration (right eye: 27.3%, left eye: 24.5%).

Serum glutamate levels were detected in all blood samples and ranged from 0.26 to 3.16 µM/µL, with a mean of 1.22 ± 0.57 µM/µL (Table 2).

Mean confocal metrics were calculated by averaging measurements from six images per eye and deriving a single overall mean across both eyes (Table 3).

### 3.2. Glutamate and Gulf War Illness

Glutamate levels were not related to symptoms of Gulf War Illness or deployment status.

### 3.3. Relationship Between Symptoms of DED, Signs of DED, and Corneal Nerve Parameters

Correlation analyses between symptom questionnaires and both clinical signs of DED and corneal nerve metrics were generally weak and inconsistent, with no robust associations identified.

### 3.4. Relationship Between Glutamate and Symptoms and Signs of DED

Glutamate levels were not associated with ocular symptoms as assessed through standardized dry eye and pain questionnaires (OSDI, DEQ5, NRS, and NPSI-Eye). Glutamate levels were, however, associated with select dry eye signs, with the strongest relationships observed for tear production and corneal staining. Higher glutamate levels were linked to reduced tear production (right eye: r = −0.05, *p* = 0.55; left eye: r = −0.25, *p* = 0.01) and greater corneal staining severity (right eye: r = 0.10, *p* = 0.29; left eye: r = 0.20, *p* = 0.03) (Table 4).

Glutamate levels were also associated with measures of nerve status, particularly corneal sensation. Higher glutamate levels corresponded to reduced corneal sensation (right eye: −0.20, *p* = 0.03; left eye: r = −0.18, *p* = 0.047) (Table 4).

### 3.5. Corneal Nerve Parameters

On confocal microscopy, higher glutamate levels were associated with reduced corneal nerve fiber width (CNFW) (r = −0.23, *p* = 0.01) but not with the remaining nerve metrics (Table 5).

### 3.6. Multivariable Modeling

Forward linear regression multivariable modeling was performed while controlling for demographics (age, sex, race, ethnicity), co-morbidities (hypertension, hyperlipidemia, diabetes mellitus, post-traumatic stress disorder, depression, arthritis, sleep apnea, benign prostatic hyperplasia), smoking status (never, former, current), and medication use (NSAIDs, aspirin, fish oil, multivitamin, beta-blocker, statin, antidepressant, antianxiety, antihistamine, sildenafil). Of the ocular signs and symptoms of DED, only corneal sensation remained significantly related to serum glutamate (β = −0.21, *p* = 0.02). Of the corneal nerve metrics, corneal nerve fiber width remained significantly related to glutamate level (β = −0.20, *p* = 0.03) after controlling for demographics (Table 6).

## 4. Discussion

In this study, we examined the relationships between serum glutamate levels, ocular symptoms, DED signs, and corneal nerve structure and function. Overall, serum glutamate showed stronger associations with corneal nerve metrics than with other clinical signs of DED. Higher serum glutamate levels were associated with alterations in corneal nerve morphology, specifically reduced corneal nerve fiber width, as well as impaired nerve function, reflected by decreased corneal sensation. In contrast, associations between higher serum glutamate levels and reduced tear production or increased corneal staining were less robust. Collectively, these findings support a broader link between systemic factors—particularly circulating glutamate—corneal nerve health and ocular surface integrity.

Our findings align with and diverge from prior research, particularly regarding the relationship between elevated glutamate levels and ocular disease pathology. One study reported significantly higher vitreous glutamate concentrations in individuals with proliferative diabetic retinopathy compared with controls—24.7 ± 14.0 μmol/L (*n* = 22) versus 9.1 ± 5.1 μmol/L (*n* = 28), *p* < 0.001 [38]—supporting a potential link between glutamate and advanced disease states in which neuropathy is a key component. This observation is consistent with our own results, where higher glutamate levels were associated with one metric of corneal nerve anatomy, namely, reduced corneal nerve fiber width. In contrast, in another study, vitreal glutamate levels were similar between individuals with and without glaucoma, 6.1 ± 2.4 μmol/L (*n* = 8) versus 5.2 ± 2.3 μmol/L (*n* = 17), *p* > 0.99 [39], though this finding should be interpreted cautiously given the limited statistical power associated with the small sample size.

Serum glutamate has also been shown to differ in systemic diseases associated with nerve dysfunction, lending credence to the possibility that similar patterns may be observed in nerve-related ocular conditions. In one study, women with chronic migraine demonstrated higher median plasma glutamate levels than controls (median [IQR]: 58.70 [44.64–72.46] (*n* = 92) vs. 38.79 [29.50–53.60] μmol/L (*n* = 50); *p* < 0.001) [40]. Systemic glutamate elevations have also been reported in several neurodegenerative diseases, including amyotrophic lateral sclerosis (163.9 ± 117.3 (*n* = 10) versus 34.1 ± 11.3 μmol/L (*n* = 10), *p* < 0.001) [41], Parkinson’s disease (71.7 ± 8.5 (*n* = 20) versus 34.1 ± 11.3 μmol/L (*n* = 20), *p* < 0.05) [42] and Alzheimer’s disease (38.47 ± 2.4 (*n* = 12) versus 27.65 ± 2.1 μmol/L (*n* = 14), *p* < 0.003) [43]. However, not all studies report elevated levels. A larger Alzheimer’s disease cohort showed significantly lower plasma D-glutamate compared with controls (785.10 ± 720.06 (*n* = 133) versus 1620.08 ± 548.80 ng/mL (*n* = 31), *p* < 0.001) [44] while a different study showed no significant difference in concentration of serum glutamate between patients with Parkinson’s disease and controls (143.1 ± 64.3 (*n* = 10) versus 124.6 ± 39.7 μM (*n* = 10)) [45]. Taken together, the literature suggests that while glutamate levels may vary in diseases involving neural dysfunction, the magnitude and direction of differences may depend on disease context, the biological source of glutamate measured, and methodological variability. In our cohort, the most robust finding was with respect to glutamate levels and nerve function, with higher glutamate levels correlating with reduced sensation.

Experimental evidence supports our clinical findings linking glutamate levels to corneal nerve anatomy and function. In an in vitro study of anoxic rat hippocampal neurons, exposure to glutamate resulted in near-complete neuronal destruction within one hour [46]. The causal role of glutamate was supported by experiments showing that co-application of gamma-D-glutamylglycine (DGG), a postsynaptic antagonist of excitatory amino acids, prevented this neuronal damage [46]. Similar findings were observed in fetal mouse neocortical cultures, in which pulsed glutamate exposure led to neuronal damage or destruction, with an 88.1 ± 2.6% reduction in visualized neurons [47]. Similar findings have been reported in human in vitro studies. In a clonal line of terminally differentiated human NT2-N neuron–like cells, a 15 min exposure to glutamate resulted in 85 ± 5% cell death within 24 h across three independent experiments [48]. These findings suggest that even short-term glutamate exposure can induce neuronal injury under experimental conditions. In addition to neuronal loss, glutamate signaling has been implicated in structural alterations of neural architecture. In an in vitro cortical neuron culture model, cells exposed to the glutamate receptor agonist *N*-methyl-D-aspartate (NMDA) for 10 min exhibited significantly fewer dendritic spine protrusions compared with cultures treated with co-application of NMDA and the NMDA receptor antagonist MK-801 (protrusion density ~1.4 vs. ~2.9, *p* < 0.01) [49]. These findings suggest that glutamate receptor activation can induce changes in neuronal morphology that are distinct from overt neuronal loss, aligning with our observation of reduced corneal nerve fiber width.

In vivo animal findings have been consistent with in vitro results. In a rat model, intravitreal injection of glutamate led to retinal neuron degeneration, quantified by immunofluorescence intensities of retinal p-tau s396 (post 1.88 ± 0.01 vs. pre 1.77 ± 0.02, *p* < 0.01) and p-tau s404 (1.87 ± 0.01 vs. 1.75 ± 0.02, *p* < 0.01), suggesting a role for glutamate in neurotoxicity to retinal cells [50]. Similarly, in a rabbit model of glaucomatous optic neuropathy, optic nerve ischemia induced by endothelin-1 was associated with increased vitreous glutamate concentrations relative to controls (264% ± 41%; *p* = 0.04) [51]. Further supporting a dose-dependent neurotoxic effect, in an in vivo study in which glutamate was delivered to the rat cortex, brain lesions consistent with excitotoxic damage increased in proportion to the amount of glutamate (Glu) administered (control ~0.5–2 mm^3^ versus 0.1 M Glu ~2–5 mm^3^, 0.5 M Glu ~15–20 mm^3^, and 1 M Glu ~28–36 mm^3^; *p* < 0.05) [52]. Taken together, in vitro and in vivo data support the association between glutamate and neurodegeneration across neural tissues both within and outside the eye and provide a plausible biological framework for the association between elevated glutamate levels and reduced corneal nerve fiber width and corneal sensitivity observed in our study.

As with all studies, several limitations should be considered when interpreting our findings. Our study population consisted predominantly of male Veterans from South Florida, which may limit generalizability to broader populations. Regarding measurements, corneal sensitivity was assessed using qualitative grading rather than quantitative esthesiometry, which reduces precision compared with other measurement modalities. Furthermore, serum glutamate levels may have been influenced by diurnal variation. Although most of our samples were collected in the morning, variability in collection timing may have contributed to measurement variability. Additionally, ocular surface inflammation (MMP-9) measurements were not obtained in a subset of subjects due to intermittent supply limitations, which may have limited evaluation of inflammatory status in relation to the primary outcomes. Regarding comorbid conditions, a majority of participants with sleep apnea reported CPAP use, which may influence ocular surface parameters. Importantly, the cross-sectional design of this study precludes assessment of temporal or causal relationships between serum glutamate levels and ocular findings, as well as evaluation of variability in measures (including IVCM) over time. Despite these limitations, our results support a model in which elevated systemic glutamate levels may be associated with structural alterations in corneal nerves, which may contribute to reduced corneal sensation and downstream disruption of tear parameters. These findings align with established neurotoxic effects of glutamate demonstrated in experimental tissue cultures, in vivo animal models, and clinical association studies. Further research is needed to extend these findings in diverse populations and determine whether modulating glutamatergic pathways may represent a therapeutic target in ocular surface disease. Future studies incorporating matched control populations and longitudinal follow-up will be important to further contextualize these findings and assess temporal relationships.

Overall, our findings are important because they suggest that systemic neurochemical status—specifically circulating glutamate—can influence ocular surface and corneal nerve health. These data support the concept that ocular diseases such as DED, which may present clinically with tear production deficits and/or ocular surface staining, can in some individuals be driven primarily by underlying nerve abnormalities rather than local inflammatory mechanisms. This distinction carries meaningful therapeutic implications, as nerve-mediated disease may require fundamentally different treatment strategies than inflammation-driven disease, even when clinical signs appear similar. Our findings also provide insight into the biological underpinnings of the discordance between DED signs and symptoms observed in prior studies [8,53]. Specifically, associations between serum glutamate levels, select clinical signs of DED, and corneal nerve morphology—but not subjective ocular symptoms—highlight the variability in patient presentations and suggest that distinct mechanisms underlie these findings. Thus, to advance the field, improved strategies are needed to identify contributors to individual DED symptoms and signs, including biochemical markers, and to better understand their biological significance. Our results suggest that glutamate may identify a neurogenic contributor to ocular surface disease, although additional studies are needed to confirm this signal. Overall, biomarkers reflecting systemic neurochemical status, corneal nerve structure and function, or their interaction are needed to enable more precise phenotyping, improve diagnostic accuracy, and guide mechanism-based treatment selection, ultimately improving quality of life for patients.

## Figures and Tables

**Table 1:** Demographics.

Demographics	Population (*n* = 124)
Age (mean ± SD)	55.67 ± 4.59 years (range 48–66)
Sex, % (*n*)	
Male	90.3% (112)
Female	9.7% (12)
Race, % (*n*)	
White	55.6% (69)
Black	41.1% (51)
Asian	3.2% (4)
Ethnicity, % (*n*)	
Hispanic	35.5% (44)
Non-Hispanic	64.5% (80)
Co-morbidities, % (*n*)	
Hypertension	38.7% (48)
Hyperlipidemia	46.0% (57)
Diabetes mellitus	16.9% (21)
Post-traumatic stress disorder	33.9% (42)
Depression	39.5% (49)
Arthritis	31.5% (39)
Sleep Apnea *	58.9% (73)
Benign Prostatic Hyperplasia	15.3% (19)
Smoking status, % (*n*)	
Never	66.1% (82)
Former	16.1% (20)
Current	16.9% (21)
Medication Use, % (*n*)	
NSAID	39.5% (49)
Aspirin	17.7% (22)
Fish Oil	22.6% (28)
Multivitamin	37.9% (47)
Beta-blocker	14.5% (18)
Statin	43.5% (54)
Antidepressant	27.4% (34)
Antianxiety	16.9% (21)
Antihistamine	29.0% (36)
Sildenafil	16.9% (21)

Abbreviations: SD = standard deviation, *n* = sample size; * Among participants with a diagnosis of sleep apnea, 75.3% reported use of Continuous Positive Airway Pressure (CPAP).

**Table 2:** Baseline Dry Eye Signs/Symptoms and Serum Glutamate.

Questionnaires, Mean ± SD	
OSDI, % ≥13	33.75 ± 23.43, 75.8%
DEQ5, % ≥6	8.93 ± 4.63, 77.5%
NRS, Right Eye	
Pain right now	1.50 ± 2.37
Average over 1 week	1.88 ± 2.34
Worst over 1 week	2.20 ± 2.69
NRS, Left Eye	
Pain right now	1.62 ± 2.46
Average over 1 week	1.89 ± 2.35
Worst over 1 week	2.15 ± 2.70
NPSI-Eye	16.33 ± 17.97
PHQ-9, % ≥5	9.96 ± 7.07, 74.3%
MFIS, % ≥38	38.05 ± 22.44, 53.3%
Ocular Surface Examination	
Inflammation (MMP-9), Right Eye, % (*n*)	
None	33.1% (41)
Mild	28.2% (35)
Moderate	11.3% (14)
Severe	4.8% (6)
Inflammation (MMP-9), Left Eye, % (*n*)	
None	29.8% (37)
Mild	30.6% (38)
Moderate	13.7% (17)
Severe	2.4% (3)
Corneal Sensation, Right Eye, % (*n*)	
Reduced	11.3% (14)
Normal	81.5% (101)
Increased	6.5% (8)
Corneal Sensation, Left Eye, % (*n*)	
Reduced	14.5% (18)
Normal	75.0% (93)
Increased	9.7% (12)
TBUT, Right Eye, mean ± SD, % ≤5	9.41 ± 4.49, 21.1%
TBUT, Left Eye, mean ± SD, % ≤5	9.76 ± 4.49, 20.3%
Corneal staining, Right Eye, mean ± SD	1.07 ± 1.89
Corneal staining, Left Eye, mean ± SD	1.14 ± 2.01
Pain before anesthetic, Right eye, mean ± SD	2.03 ± 2.77
Pain after anesthetic, Right eye, mean ± SD, % persistent pain	0.78 ± 1.75, 27.3%
Pain before anesthetic, Left eye, mean ± SD	1.87 ± 2.69
Pain after anesthetic, Left eye, mean ± SD, % persistent pain	0.73 ± 1.68, 24.5%
Schirmer score, Right eye, mean ± SD	15.7 ± 9.62
Schirmer score, Left eye, mean ± SD	16.31 ± 9.55
IOP, Right Eye, mean ± SD	16.89 ± 3.21
IOP, Left Eye, mean ± SD	16.71 ± 3.24
Serum Glutamate, mean ± SD (µM/µL)	1.22 ± 0.57

Abbreviations: SD = standard deviation, *n* = sample size, OSDI = Ocular Surface Disease Index, DEQ5 = 5-Item Dry Eye Questionnaire, NRS = Numerical Rating Scale, NPSI-Eye = Neuropathic Pain Symptom Inventory, modified for Eye pain, PHQ-9 = Patient Health Questionnaire, MFIS = Modified Fatigue Impact Scale, MMP-9 = matrix metalloproteinase 9, TBUT= tear break up time, IOP = Intraocular pressure.

**Table 3:** Corneal Nerve Metrics.

Corneal Nerve Metrics, Mean ± SD	
Corneal nerve fiber density (CNFD; fibers/mm^2^)	15.20 ± 9.06
Corneal nerve fiber length (CNFL; mm/mm^2^)	10.66 ± 4.27
Corneal nerve branch density (CNBD; branches/mm^2^)	18.90 ± 16.2
Corneal nerve fiber area (CNFA; mm^2^/mm^2^)	0.01 ± 0.01
Corneal nerve fiber width (CNFW; mm/mm^2^)	0.02 ± 0.003
Corneal total branch density (CTBD; branches/mm^2^)	32.54 ± 20.86
Corneal nerve fractal dimension	1.44 ± 0.06

**Table 4:** Correlations between Ocular Signs/Symptoms and Serum Glutamate.

	r	*p*-Value
Questionnaires		
OSDI	−0.09	0.35
DEQ5	−0.01	0.92
NRS, Right Eye		
Pain right now	−0.03	0.78
Average over 1 week	−0.03	0.71
Worst over 1 week	−0.03	0.72
NRS, Left Eye		
Pain right now	0.03	0.73
Average over 1 week	0.01	0.94
Worst over 1 week	0.03	0.73
NPSI-Eye	−0.02	0.80
Ocular Surface		
Inflammation (MMP-9), Right Eye	−0.04	0.73
Inflammation (MMP-9), Left Eye	0.16	0.13
Corneal Sensation, Right Eye	−0.20	0.03 *
Corneal Sensation, Left Eye	−0.18	0.05 *
TBUT, Right Eye	−0.02	0.86
TBUT, Left Eye	0.02	0.87
Corneal staining, Right Eye	0.10	0.29
Corneal staining, Left Eye	0.20	0.03 *
Pain before anesthetic, Right eye	−0.15	0.09
Pain after anesthetic, Right eye	0.00	1.00
Pain before anesthetic, Left eye	−0.15	0.11
Pain after anesthetic, Left eye	−0.02	0.80
Schirmer score, Right eye	−0.05	0.55
Schirmer score, Left eye	−0.25	0.01 *
IOP, Right Eye	0.02	0.80
IOP, Left Eye	0.06	0.50

Abbreviations: r = correlation coefficient, *p*-value = probability value, OSDI = Ocular Surface Disease Index, DEQ5 = 5-Item Dry Eye Questionnaire, NRS = Numerical Rating Scale, NPSI-Eye = Neuropathic Pain Symptom Inventory, modified for Eye pain, MMP-9 = matrix metalloproteinase 9, TBUT= tear break up time, IOP = Intraocular pressure, * *p* < 0.05.

**Table 5:** Correlations between Corneal Nerve Metrics and Serum Glutamate.

	r	*p*-Value
Corneal nerve fiber density (CNFD; fibers/mm^2^)	0.11	0.24
Corneal nerve fiber length (CNFL; mm/mm^2^)	0.07	0.43
Corneal nerve branch density (CNBD; branches/mm^2^)	0.14	0.12
Corneal nerve fiber area (CNFA; mm^2^/mm^2^)	−0.01	0.89
Corneal nerve fiber width (CNFW; mm/mm^2^)	−0.23	0.01 *
Corneal total branch density (CTBD; branches/mm^2^)	0.09	0.34
Corneal nerve fractal dimension	0.06	0.50

Abbreviations: * represents *p* < 0.05.

**Table 6:** Multivariable Models.

Model	Standardized Coefficients (β)	*p*-Value
Sensation	−0.21	0.02 *
Corneal Nerve Fiber Width (CNFW)	−0.20	0.03 *

Abbreviations: * *p* < 0.05.

## Data Availability

The original contributions presented in this study are included in the article. Further inquiries can be directed to the corresponding author.
